# Gut tumors in flies alter the taste valence of an anti-tumorigenic bitter compound

**DOI:** 10.1016/j.cub.2024.07.021

**Published:** 2024-07-16

**Authors:** Nicole Y. Leung, Chiwei Xu, Joshua Shing Shun Li, Anindya Ganguly, Geoff T. Meyerhof, Yannik Regimbald-Dumas, Elizabeth A. Lane, David T. Breault, Xi He, Norbert Perrimon, Craig Montell

Since publication, we have identified an error in Figure S2. We mistakenly inserted a duplicated version of Figure S2D into Figure S2B. Figure S2B has now been updated online. This error does not affect the figure legends, texts, or conclusions of the paper. We apologize for this error and any confusion it may have caused.

## Figures and Tables

**Figure S2B.**
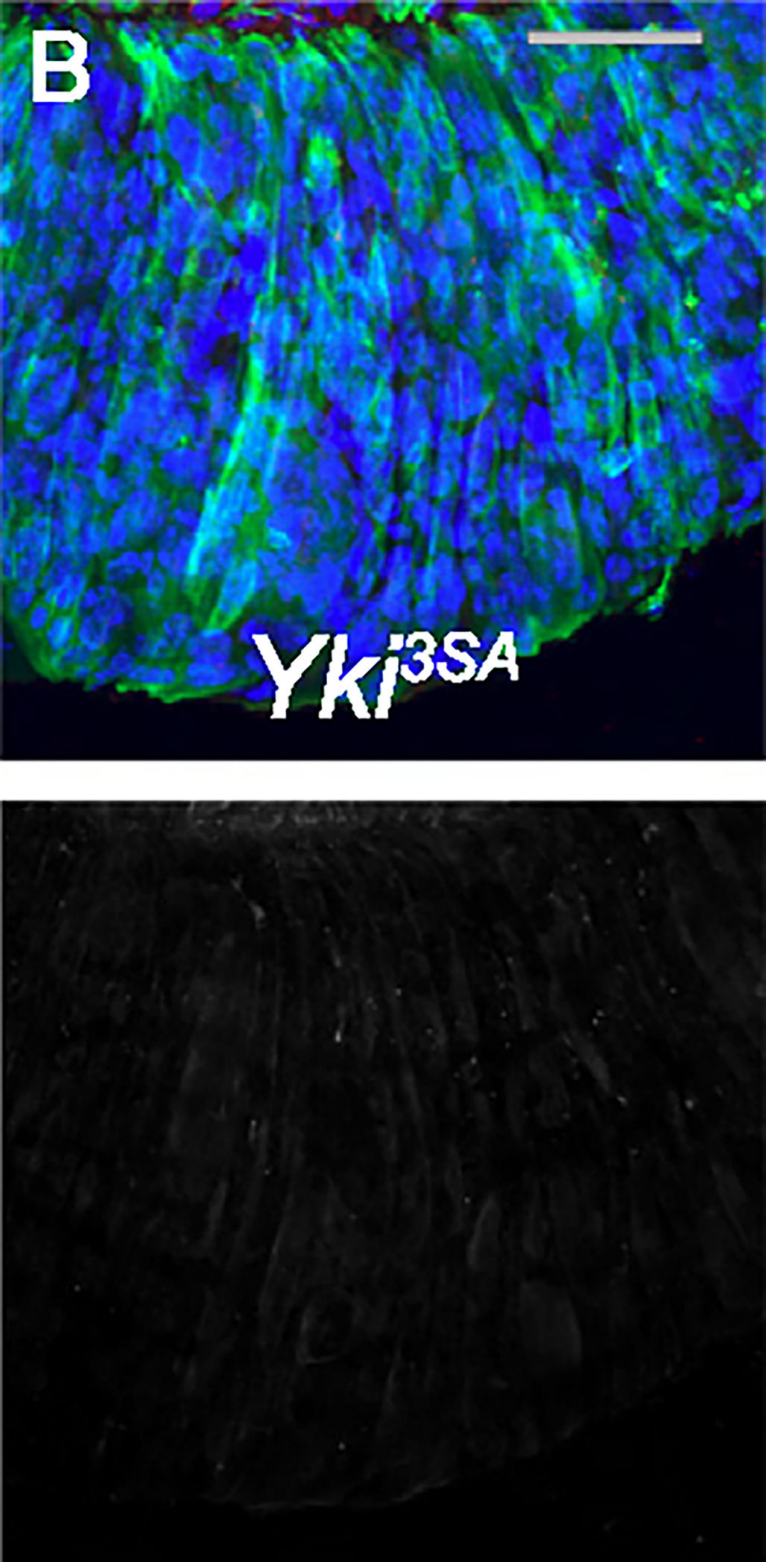
ARI inhibits fly gut tumors by inducing ISC differentiation and apoptosis, Related to Figures 2–4. (corrected)

**Figure S2B.**
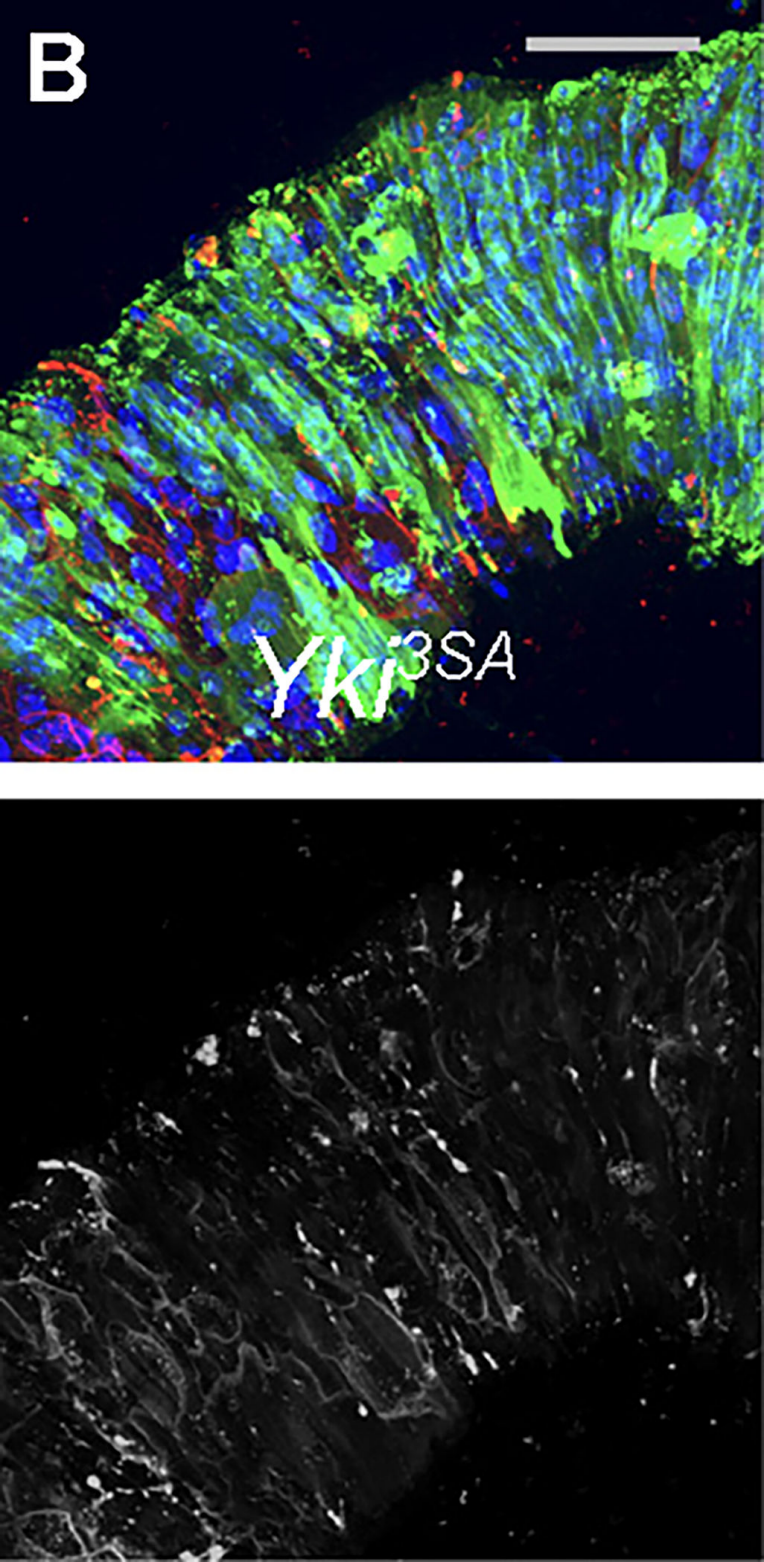
ARI inhibits fly gut tumors by inducing ISC differentiation and apoptosis, Related to Figures 2–4. (original)

